# Macrofauna associated with temporary *Sabellaria alveolata* reefs on the west coast of Cotentin (France)

**DOI:** 10.1186/s40064-016-2885-y

**Published:** 2016-08-05

**Authors:** Erika Schlund, Olivier Basuyaux, Billie Lecornu, Jean-Philippe Pezy, Alexandrine Baffreau, Jean-Claude Dauvin

**Affiliations:** 1 UNICAEN, UNIROUEN, UMR M2C, CNRS 6143, Normandie Univ, 24 Rue des Tilleuls, 14000 Caen, France; 2 CREC, Station Marine, Université de Caen Normandie, 54 Rue du Docteur Charcot, BP 49, 14530 Luc-sur-Mer, France; 3 SMEL, Centre Expérimental, ZAC Blainville-sur-Mer, 50560 Blainville-sur-Mer, France

**Keywords:** *Sabellaria* reefs, Macrofauna taxonomic richness, Bay of Mont-Saint-Michel, Cotentin coast, Temporary reefs

## Abstract

The polychaete *Sabellaria alveolata* (Linnaeus, 1767) is an important ecosystem engineer building reef structures which add to the topographic complexity in colonized areas. In Europe, the most extensive reef formation is located in the Bay of Mont-Saint-Michel (France). Since 2006, *Sabellaria* bio-constructions have developed on hard substrates along the west coast of the Cotentin Peninsula between Champeaux and Saint-Germain-sur-Ay on the northern part of the Bay of Mont-Saint-Michel. In this sector, two distinct types of bio-construction can be distinguished: platforms and reefs. The aim of this study is to analyse the patterns of the associated macrofauna on these platforms and reefs, as well as outside, and test for a correlation between the presence of *Sabellaria* bio-constructions and the richness of the benthic macrofauna. Univariate analyses are used to compare the macrofauna on four sites (Champeaux, Lingreville, Blainville-sur-Mer and Saint-Germain-sur-Ay). The results show a higher taxonomic richness on the platform-type than on the reef-type structures, and also a higher taxonomic richness outside the bio-construction areas. This suggests that, on the examined sites, the presence of *S. alveolata* bio-constructions does not contribute to higher levels of benthic macrofaunal richness on hard substrates. Temporary bio-constructions along this coast exhibit reefs of interest at some sites as well as in very small zones which merit special attention.

## Background

Biogenic structures built by ecosystem engineers such as corals, molluscs and polychaetes provide favourable habitats for other benthic invertebrates and vertebrates; moreover, these structures can play an important role as nurseries for certain species of commercial interest and also represent an essential source of food for birds and fishes (Commito and Rusignuolo 2000). In Europe, ‘reefs’ are recognized as marine habitats to be protected and are listed under Annex I of the EU Habitats Council Directive 92/43/EEC under the Habitat Code 1170 (Reefs). The habitat ‘*Sabellaria alveolata* Reefs’ also benefits from a Biodiversity Action Plan in the UK (http://www.jncc.gov.uk/page-5155).

**Fig. 1:**
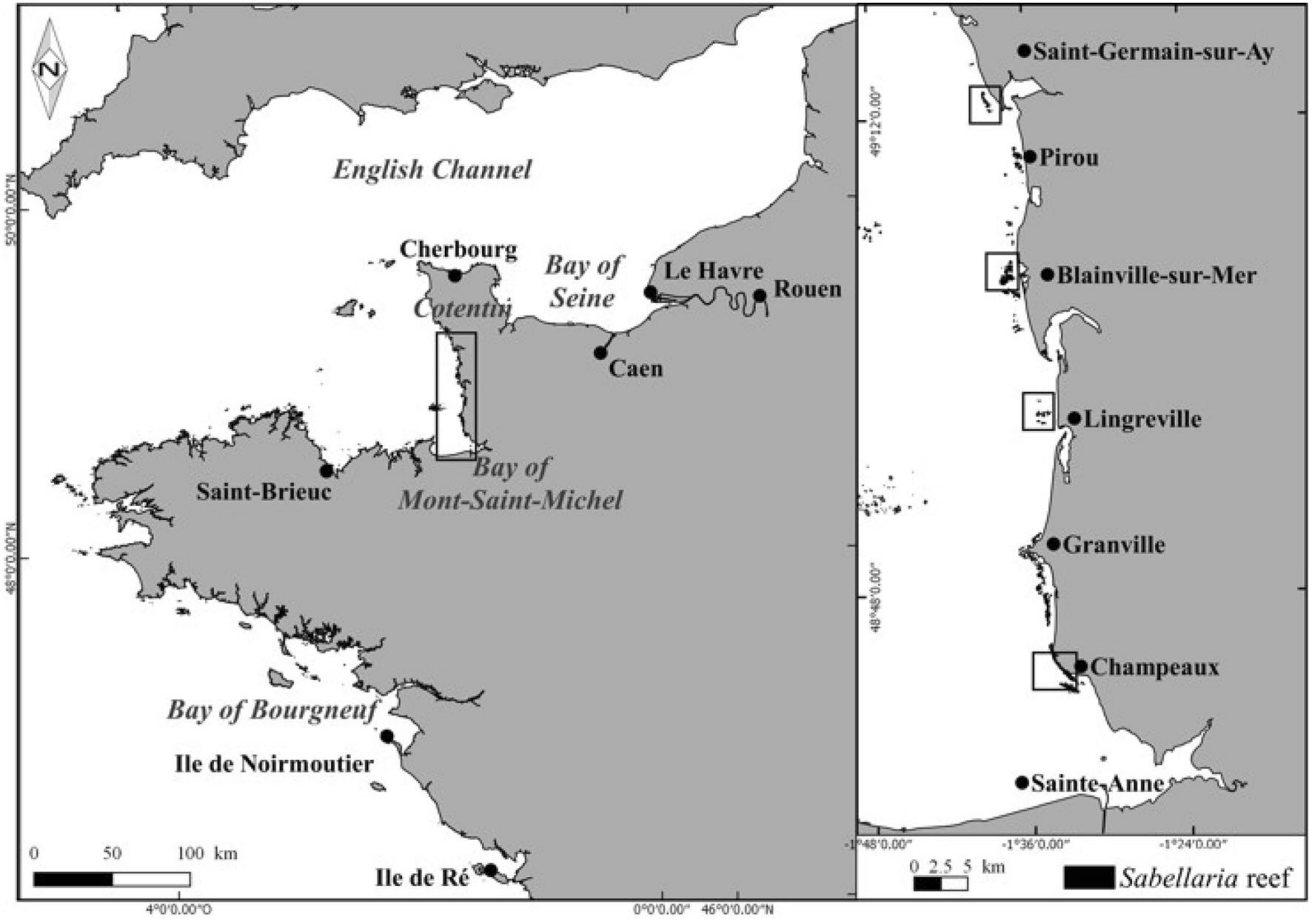
Location of study sites on the west coast of Cotentin, France, western part of the English Channel and location of the Bay of Bourgneuf along the Atlantic coast

The honeycomb worm *S. alveolata*, which belongs to the family Sabellaridae, is present in temperate regions of the world (India, North and South America), and is also known in Europe, where it ranges from the Bristol Channel (Wilson 1974) to the coast of Portugal (Dias and Paula 2001). The bio-constructions correspond to polychaete colonies which build up sediment agglomerates composed of tubes. The initial tubes bind to the rock in the mid-intertidal zone, thus forming structures called platforms. These platforms develop throughout the life of the individuals, eventually creating more massive structures—referred to as reefs—in up to 3–5 years (Gruet 1982). In France (Fig. 1), the major bio-constructions in the Bay of Bourgneuf (Vendée, on the Atlantic coast) and in the Bay of Mont-Saint-Michel (bordering the western basin of the English Channel) represent the two largest structures of this type in Europe (Gruet and Bodeur 1997). *Sabellaria* reefs are also developed on sand flats in the lower intertidal zone of the Bay of Mont-Saint-Michel, where they form the Saint-Anne reef (Dubois et al. 2002, 2006).

The examination of numerous formations by Gruet (1972a, 1982) has provided a basis for distinguishing different phases of reef evolution. The natural evolution of a reef begins with the settlement of young recruits building up ball-shaped structures which then coalesce as they grow to form barriers; this is followed by destruction of the barriers, due to sedimentation, colonization by epibionts (mainly mussels or oysters) or extreme hydrodynamic conditions during storms (Gruet and Bodeur 1997; Dubois et al. 2006).

Environmental conditions (stable temperature, humid atmosphere, reduced light, etc.) favourable for marine macrofauna living in crevices are created during the phases of construction and destruction of a reef, i.e. growth, degradation, death and colonization (Gruet 1970, 1971, 1972a, b, 1977, 1981, 1982). Gruet (1971, 1982) and Gruet and Bodeur (1997) stressed that the Taxonomic Richness (TR) of the associated benthic macrofauna decreases in areas with high densities of the honeycomb worm (15,000–60,000 ind m^−2^), and observed that TR is higher in degraded reef zones. The *S. alveolata* bio-constructions of the Sainte-Anne reef in the southern Bay of Mont-Saint-Michel have a high TR compared with the very low TR observed in the surrounding intertidal soft-bottom *Macoma balthica* community (Dubois et al. 2002, 2006). In the Mediterranean Sea, both in the shallow waters of the Valencia Gulf (Spain; Porras et al. 1996) and in deeper waters of the Tyrrhenian Sea (Italy; La Porta and Nicoletti 2009), high polychaete richness is reported to be associated with *S. alveolata* reefs, especially during the reef destruction phase. Anadon (1981) described the associated fauna in two reefs of the Ria de Vigo (Galicia, Spain), which show a similar pattern to that observed in Gruet’s studies: i.e. a lower TR in areas with a high population density of the honeycomb worm. Dias and Paula (2001) described the associated fauna in two intertidal *S. alveolata* reefs from the central coast of Portugal, with two main patterns, i.e. crustaceans are dominant at the Magoita site and polychaetes at the Avencas site.

The modelling of larval dispersal (Ayata et al. 2009) shows that *S. alveolata* living on the western coast of Cotentin are unlikely to participate in renewal of the populations. Therefore, along the west coast of Cotentin north of Champeaux, the temporary *Sabellaria* structures (established for probably less than 10 years) are associated with a southerly source of larvae coming from populations at Saint-Anne and Champeaux in the Bay of Mont-Saint-Michel. In certain years, *Sabellaria* larvae can reach the studied sites owing to south-westerly winds plus the lower velocity of nearshore tidal currents (Ayata et al. 2009).

A considerable development of *Sabellaria* was observed on the west coast of Cotentin in the late years 2000. This development was particularly marked on the mid-littoral bedrock zone colonized by the macroalgae *Fucus serratus,* which can produce changes in habitat and functional modifications due to the establishment of high densities of the honeycomb worm *S. alveolata*.

The aim of this study is to assess the macrofauna richness associated with three types of habitat (*S. alveolata* platform, reef and outside reef) of the mid-intertidal zone at four sites (Champeaux, Lingreville, Blainville-sur-Mer and Saint-Germain-sur-Ay) located on the west coast of Cotentin, taking into account the northward expansion of the reefs in the years 2000.

## Methods

### Study area

The study area is located on the west coast of Cotentin, between Champeaux (in the south) and Saint-Germain-sur-Ay (in the north), in a sector where the first recent *Sabellaria alveolata* bio-constructions were observed in 2006 (Delhay 2010; Basuyaux 2011) (Fig. 1). In the intertidal zone of this highly dynamic coastline, bio-constructions show a discontinuous development since the hard substrate is interspersed with soft-bottom areas mainly composed of sand and gravel. Using the observations carried out in 2010–2011 (Basuyaux 2011), four sampling sites were selected in 2014 within the study area on the West Cotentin coast [i.e. from south to north: Champeaux (CHAM), Lingreville (LING), Blainville-sur-Mer (BLSM) and Saint-Germain-sur-Ay (SGSA)] to study the TR associated with the *Sabellaria* bio-constructions. In addition, except for CHAM, the selected sites are all located near deltaic estuaries with high-energy hydrodynamic conditions which induce sediment displacement on this wave-dominated shore (Beck et al. 2015).

*Sabellaria* reef formations were observed in the early 1960 s at a location north of Blainville-sur-Mer (Fig. 1) by Hommeril (1967). These reefs were affected by destruction just after the severe winter of 1962–1963 due the high sensitivity of *S. alveolata* to low temperatures (Hommeril and Larsonneur 1963). Later, mapping of the intertidal benthic communities carried out in 1982–1984 along the western coast of Cotentin (Guillaumont et al. 1987) identified a single small reef at Pirou to the south of Saint-Germain-sur-Ay as well as some fragmented reefs south of Granville extending as far as Champeaux. Since 2006, fragmented *Sabellaria* reef structures have been observed along the west coast of Cotentin in the north of the Bay of Mont-Saint-Michel, between Champeaux and Saint-Germain-sur-Ay (Fig. 1). In 2010–2011, these bio-constructions occupied an area of 2.28 km^2^, divided into 535 more or less extensive patches between Champeaux and Saint-Germain-sur-Ay along ~60 km of coastline (Basuyaux 2011).

The bio-constructions are fragmented into a large number of units, but their generalorientation is parallel to the coast (Basuyaux personal observations), which is also mainly parallel to the prevailing tidal current (i.e. NW–SW sector) as in the Bay of Mont-Saint-Michel (Dubois et al. 2002). The distinctive feature of bio-constructions at the studied sites is that they are developed on hard substrates, except at CHAM where they develop on soft substrate (O. Basuyaux and J. C. Dauvin, personal observations). In the present study, bio-constructions are classified into two main groups: platforms and reefs; i.e. the platform type comprises bio-constructions which do not exceed 30 cm in height, while the reef type includes bio-constructions higher than 30 cm.

### Field and laboratory procedures

Sampling was carried out on the four sites between February and April 2014 (on 18 February for BLSM, 3 March for CHAM, 18 March for SGSA and 31 March for LING (Table 1), using a circular corer of diameter of 0.20 m (about 1/32 m^2^). Cores were burrowed into the reefs or the platforms as deeply as possible (mainly between 0.10 and 0.30 m depending on the height of the bio-construction). For each site, sixteen cores were sampled; eight from a platform zone and eight from a reef zone, making up a total of 64 cores. The collected sediment was preserved in 10 % formalin.

**Table 1:** Sampling strategy in 2014

Site	WGS 84 coordinates	Sampling dates	Sample strategy
CHAM	48°43′52ʺN-1°33′25ʺW	3 March	Cores (Spatial comparison)
		28 April	Quadrats
LING	48°57′074ʺN-1°34′429ʺW	31 March	Cores (Spatial comparison)
BLSM	49°4′7ʺN-1°37′25ʺW	18 February	Cores (Spatial comparison)
		18 June and 18 August	Cores (Temporal comparison)
		16 April	Quadrats
SGSA	49°12′645ʺN-1°38′693ʺW	18 March	Cores (Spatial comparison)
		17 April	Quadrats

*CHAM* Champeaux, *LING* Lingreville, *BLSM* Blainville-Sur Mer, *SGSA* Saint-Germain-Sur-Ay

To study the temporal changes in TR at BLSM during the winter–summer period, two other samplings were carried out on both platforms and reefs on 18 June and 18 August 2014, making a total of 32 additional cores (Table 1).

In the laboratory, the *S. alveolata* tubes were disaggregated in seawater, and the macrofauna retained on a 500 µm mesh size was sorted, counted and identified to be lowest taxonomic level. Nematodes and other meiofauna were excluded from the analyses because the sorting method—i.e. mesh size—was unsuitable (Dubois et al. 2002).

A second sampling strategy was performed to compare the TR of areas with *S. alveolata* reefs with areas of hard substrate without any bio-constructions located at the same elevation in the intertidal zone but about 100 m away from the reefs. This strategy was applied to the permanent reef at CHAM, as well as to BLSM and SGSA where the reefs remained well developed in 2014. The samples were collected on 16 April (BLSM) and 17 April (SGSA), and 28 April (CHAM), by scraping to 0.05 m depth over a quadrat surface-area of 0.1 m^2^. For the three sites, ten quadrats representing a total area of 1 m^2^ were sampled from a reef zone and from outside the reef structure on hard substrate. Thus, a total of 60 quadrats were collected from the three sampling sites, the samples being preserved, sieved on 0.5-mm mesh and identified in the same way as for the core samples.

### Data analysis

Macrofauna data are analysed by combining species richness [after updating the species name and synonymy using WORMS (World Register of Marine Species): http://www.marinespecies.org; accessed on 15 December 2015] and Hill’s diversity numbers (Hill 1973) as recommended by Heip et al. (1988): N1 = exp (H’) with H’ is the Shannon-Wiener diversity (Shannon 1948); and N2 = 1/SI, with SI is the Simpson’s dominance index (Simpson 1949).

A Shapiro–Wilk normality test and a Bartlett’s test for homogeneity of variance are performed prior to each ANOVA with the R software to validate the assumptions of ANOVA. Then, ANOVAs are performed to assess:

- the spatial and temporal abundance patterns of *S. alveolata* on reef and platform structures (core sampling);
- the spatial and temporal patterns of associated macrofauna (TR and total abundances) on reef and platform structures (core sampling);
- the TR inside and outside reef structures (quadrat sampling; TR and total abundances).

A Tukey Honestly Significant Difference test is used to determine differences between the sites, types of structure (reef or platform) and sampling dates.

To compare the faunal TR in the three communities, i.e. from the platform, from the reef or outside the reef area*, k*-dominance curves are plotted for each substratum type and site and the associated species, excluding *S. alveolata*, are ranked in order of their dominance in terms of abundance.

## Results

### *Spatial pattern of* Sabellaria alveolata *abundance (core sampling)*

**Fig. 2:**
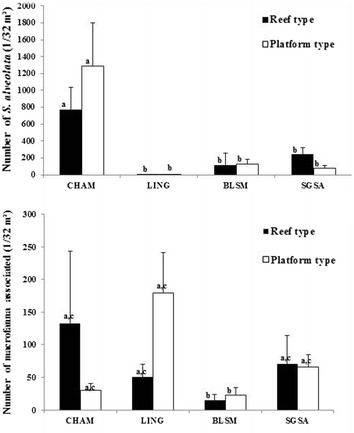
Density of *Sabellaria alveolata* and other species (number of individuals on 1/32 m^2^ and Standard Deviation). *CHAM* Champeaux, *LING* Lingreville, *BLSM* Blainville-Sur-Mer, *SGSA* Saint-Germain-Sur-Ay (core sampling). Means with the same superscript do not differ significantly (Tukey’s HSD test; *p* > 0.05)

*Sabellaria alveolata* is the most abundant species regardless of the site location or substrate type, apart from LING, where it does not appear among the dominant species and shows lower abundance on the degraded reef (Tables 2, 3). Comparison between the two different structures (platform and reef) do not reveal significant differences (ANOVA F_1.56_ = 2.96; *p* = 0.09), but there is a significant difference in the densities of *S. alveolata* between different sites (ANOVA F_3.56_ = 7.55; *p* < 0.01). The highest densities of *S. alveolata* are observed in the CHAM population (Tukey test) (Fig. 2).

**Table 2:** Relative percentage of *Sabellaria alveolata* density against total density at each site for sampling dates in 2014 (core sampling)

	February–March		June		August	
	Platform	Reef	Platform	Reef	Platform	Reef
CHAM	98	85	–	–	–	–
LING	1	0	–	–	–	–
BLSM	94	89	85	81	78	31
SGSA	55	77	–	–	–	–

*CHAM* Champeaux, *LING* Lingreville, *BLSM* Blainville-Sur-Mer and, *SGSA* Saint-Germain-Sur-Ay

**Table 3:** Top-ranked species densities (core sampling, mean per 1/32 m^2^ ± SD) of macrofauna in *Sabellaria alveolata* bio-constructions

Site	Rank	Platform type		Reef type	
		Taxa	Mean density ± SD	Taxa	Mean density ± SD
Saint-Germain-sur-Ay	1	*Sabellaria alveolata*	80.00 ± 31.21	*Sabellaria alveolata*	242.75 ± 77.12
	2	*Golfingia (Golfingia) vulgaris*	16.13 ± 6.77	*Porcellana platycheles*	32.50 ± 30.16
	3	*Porcellana platycheles*	11.88 ± 14.79	*Golfingia (Golfingia) vulgaris*	14.13 ± 11.57
	4	*Notomastus latericeus*	6.63 ± 6.30	*Cirratulus cirratus*	2.63 ± 7.42
	5	*Gibbula umbilicalis*	5.75 ± 3.41	Nemertea	1.88 ± 1.73
Blainville-sur-Mer	1	*Sabellaria alveolata*	130 ± 53.86	*Sabellaria alveolata*	115.75 ± 140.50
	2	*Golfingia (Golfingia) vulgaris*	12.25 ± 8.41	*Gibbula umbilicalis*	7.13 ± 7.85
	3	*Acanthochitona crinita*	1.25 ± 1.39	*Porcellana platycheles*	2.13 ± 2.23
	4	*Perinereis cultrifera*	1.13 ± 1.81	*Golfingia (Golfingia) vulgaris*	1.25 ± 1.49
	5	*Spirobranchus triqueter*	1.00 ± 1.07	*Perinereis cultrifera*	1.00 ± 1.41
Lingreville	1	*Porcellana platycheles*	17.25 ± 26.72	*Sphaeroma serratum/monodi*	24.38 ± 10.32
	2	Nemertea	4.75 ± 3.54	*Porcellana platycheles*	7.88 ± 13.25
	3	*Mytilus edulis*	3.13 ± 2.47	Nemertea	5.88 ± 13.04
	4	*Gibbula umbilicalis*	2.25 ± 2.82	*Gibbula umbilicalis*	3.00 ± 1.85
	5	*Malacoceros fuliginosus*	1.75 ± 2.19	*Golfingia (Golfingia) vulgaris*	1.75 ± 2.38
Champeaux	1	*Sabellaria alveolata*	1182.88 ± 676.30	*Sabellaria alveolata*	771.88 ± 265.52
	2	*Eulalia ornata*	5.63 ± 4.27	*Sphaeroma serratum/monodi*	100.13 ± 66.25
	3	*Perinereis cultrifera*	3.75 ± 4.03	*Porcellana platycheles*	15.75 ± 37.17
	4	*Malacoceros fuliginosus*	2.75 ± 4.30	*Golfingia (Golfingia) vulgaris*	5.38 ± 9.53
	5	*Sphaeroma serratum/monodi*	2.63 ± 5.07	Actiniaria	3.50 ± 5.40

See “Appendix 1” for taxonomic details and complete species list

### *Temporal pattern of* Sabellaria alveolata *abundance at BLSM (core sampling)*

**Fig. 3:**
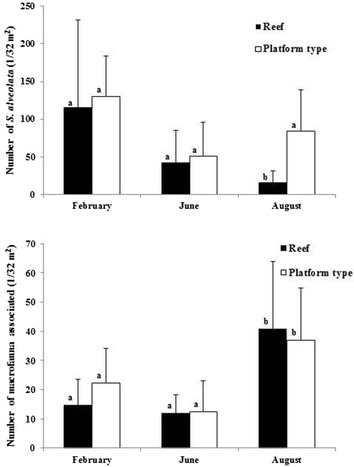
Temporal changes in density of *Sabellaria alveolata* and other species (number of individuals on 1/32 m^2^ and Standard Deviation) according to the different sampling dates at Blainville-sur-Mer (core sampling). Means with the same superscript do not differ significantly (Tukey’s HSD test; *p* > 0.05)

As observed with the spatial pattern, *Sabellaria alveolata* at BLSM exhibits higher densities than the other species over time (Table 4; see also “Appendix 2”). However, from February to August, the density of *S. alveolata* shows a clear decrease on both platform and reef stations (Fig. 3); a significant difference of *S. alveolata* abundance appears between winter (February), when the maximum occurs, and summer (August) (ANOVA F_2,42_ = 21.96; *p* < 0.001) (Tukey test).

**Table 4:** Top-ranked species densities (core sampling, mean per 1/32 m^2^ ± SD) of macrofauna in *Sabellaria alveolata* bio-constructions on different dates at Blainville-Sur-Mer

Date	Rank	Platform type		Reef type	
		Species	Mean density ± SD	Species	Mean density ± SD
February	1	*Sabellaria alveolata*	130.00 ± 53.86	*Sabellaria alveolata*	115.75 ± 140.50
	2	*Acanthochitona crinita*	1.25 ± 1.39	*Gibbula umbilicalis*	7.13 ± 7.85
	3	*Perinereis cultrifera*	1.13 ± 1.81	*Porcellana platycheles*	2.13 ± 2.23
	4	*Spirobranchus triqueter*	1.00 ± 1.07	*Perinereis cultrifera*	1.00 ± 1.41
	5	*Platynereis dumerilii*	0.88 ± 0.99	*Venerupis corrugata*	0.63 ± 0.52
June	1	*Sabellaria alveolata*	51.63 ± 44.42	*Sabellaria alveolata*	42.63 ± 38.63
	2	*Porcellana platycheles*	2.88 ± 5.36	*Gibbula umbilicalis*	6.50 ± 3.66
	3	*Gibbula umbilicalis*	2.00 ± 1.60	*Venerupis corrugata*	0.75 ± 1.03
	4	*Dynamene bidentata*	1.50 ± 1.60	*Porcellana platycheles*	0.63 ± 1.06
	5	*Eumida sanguinea*	0.50 ± 1.07	*Eumida sanguinea*	0.50 ± 0.53
August	1	*Sabellaria alveolata*	84.38 ± 54.22	*Sabellaria alveolata*	16.00 ± 16.09
	2	*Gibbula umbilicalis*	4.88 ± 4.36	*Gibbula umbilicalis*	12.38 ± 12.83
	3	*Platynereis dumerilii*	3.25 ± 4.89	*Cyathura carinata*	8.38 ± 6.16
	4	*Carcinus maenas*	2.50 ± 2.51	*Porcellana platycheles*	4.38 ± 3.25
	5	*Notomastus latericeus*	2.38 ± 1.69	*Perinereis cultrifera*	2.75 ± 1.49

See “Appendix 2” for taxonomic details and complete species list

As observed with the spatial pattern, the temporal pattern of *Sabellaria alveolata* does not differ significantly between platform and reef structures (ANOVA F_1.42_ = 0.004; *p* = 0.94) (Fig. 3).

### Spatial pattern of associated macrofauna on platform and reef structures (core sampling)

From the 64 cores, a total of 6731 individuals are identified (67 to species level) belonging to 80 taxa. The richest group is the polychaetes with 36 taxa. Although other species show much lower abundances than *S. alveolata*, it is noteworthy that their abundances are higher on the reefs than on the platforms.

**Fig. 4:**
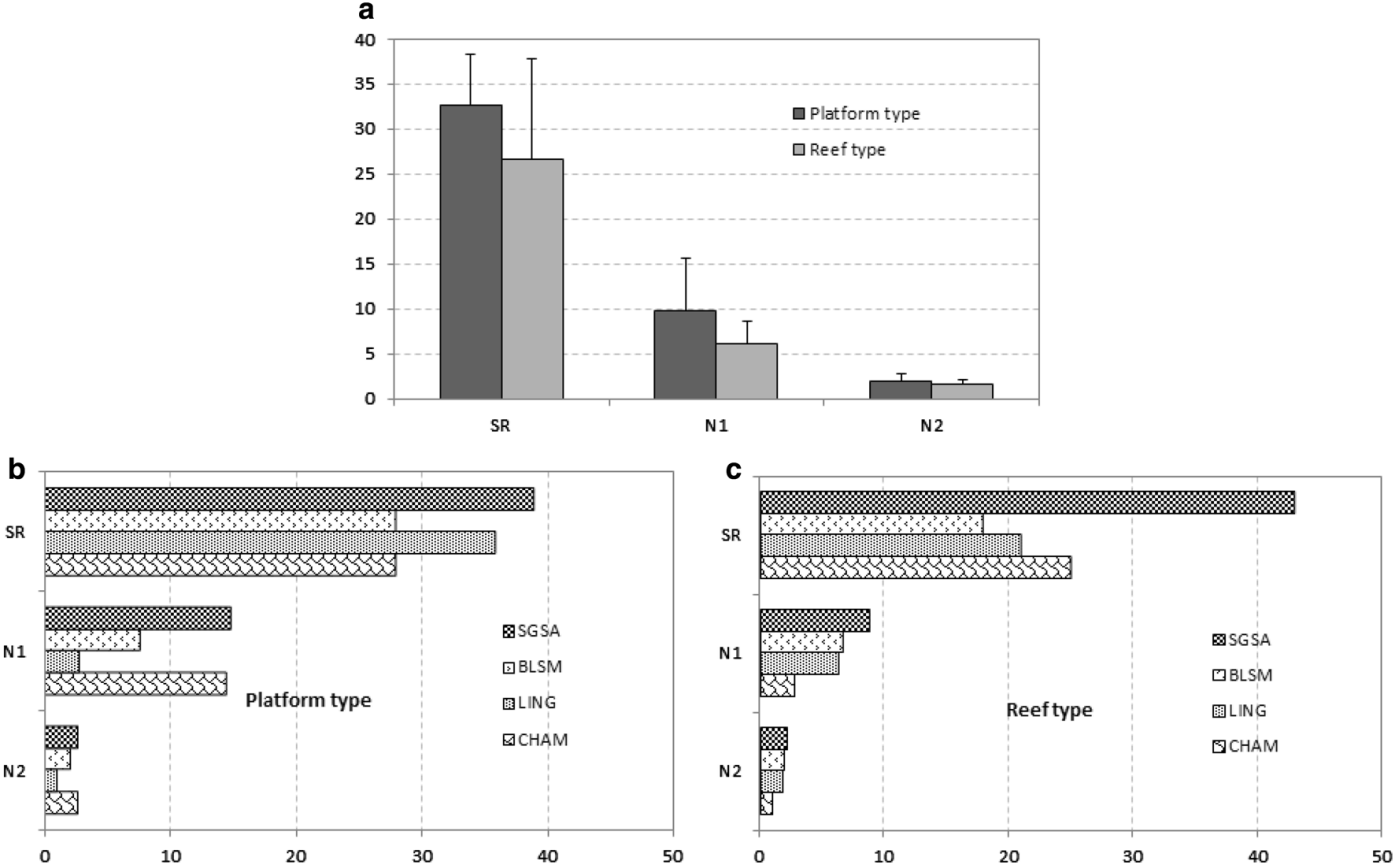
**a** Values of species richness (SR) and Hill’s numbers (N1, N2) for platform and reef-type structures (core sampling); **b, c** values of SR, N1 and N2 for platform and reef-type structures (values excluding *S. alveolata). CHAM* Champeaux, *LING* Lingreville, *BLSM* Blainville-Sur-Mer, *SGSA* Saint-Germain-Sur-Ay

The taxonomic richness is significantly different between the reefs and platforms and also between different sites (Table 5). Moreover, the interactions between the two different structures and their locations are not significantly different (Table 5). The Tukey test reveals that the CHAM and LING sites show the greatest differences in TR between reefs and platforms (Table 5; Fig. 4).

**Table 5:** Results of Two-way ANOVA on taxonomic richness and total abundance values for the spatial pattern of associated macrofauna on platform and reef structures (core sampling)

	Taxonomic richness			Total abundance		
	Df	F	*p*	Df	F	*p*
Reef/platform (A)	1	6.46	<0.05	1	0.36	0.55
Site (B)	3	10.89	<0.01	3	10.69	<0.01
A × B	3	0.09	0.97	3	15.10	<0.01
∑	8			8		

F: value of the Fisher law; *p* : probability of the factor or interaction; A = stations sampled in two structures; B = samples made at four sites; (A × B) = interaction between factor A and B

Site location is a significant factor influencing abundance, and the interactions between the two different structures and location are significantly different (Table 5). The SGAA and BLSM sites have lower macrofauna abundances compared with CHAM or LING (Tukey test).

The faunal composition for the CHAM site differs from that observed for BLSM and SGSA. If we exclude *S. alveolata*, two groups dominate at these three sites (CHAM, BLSM and SGSA). Arthropods are represented by 18 taxa, including two dominant species: the decapod *Porcellana platycheles* and the isopods *Sphaeroma* spp. Mollusca are represented by 18 taxa, with the gastropod *Gibbula umbilicalis* as dominant species (Table 3; see also “Appendix 1”). The sipunculid *Golfingia vulgaris* is also present among the five top-ranking species on all four sites (Table 3).

**Fig. 5:**
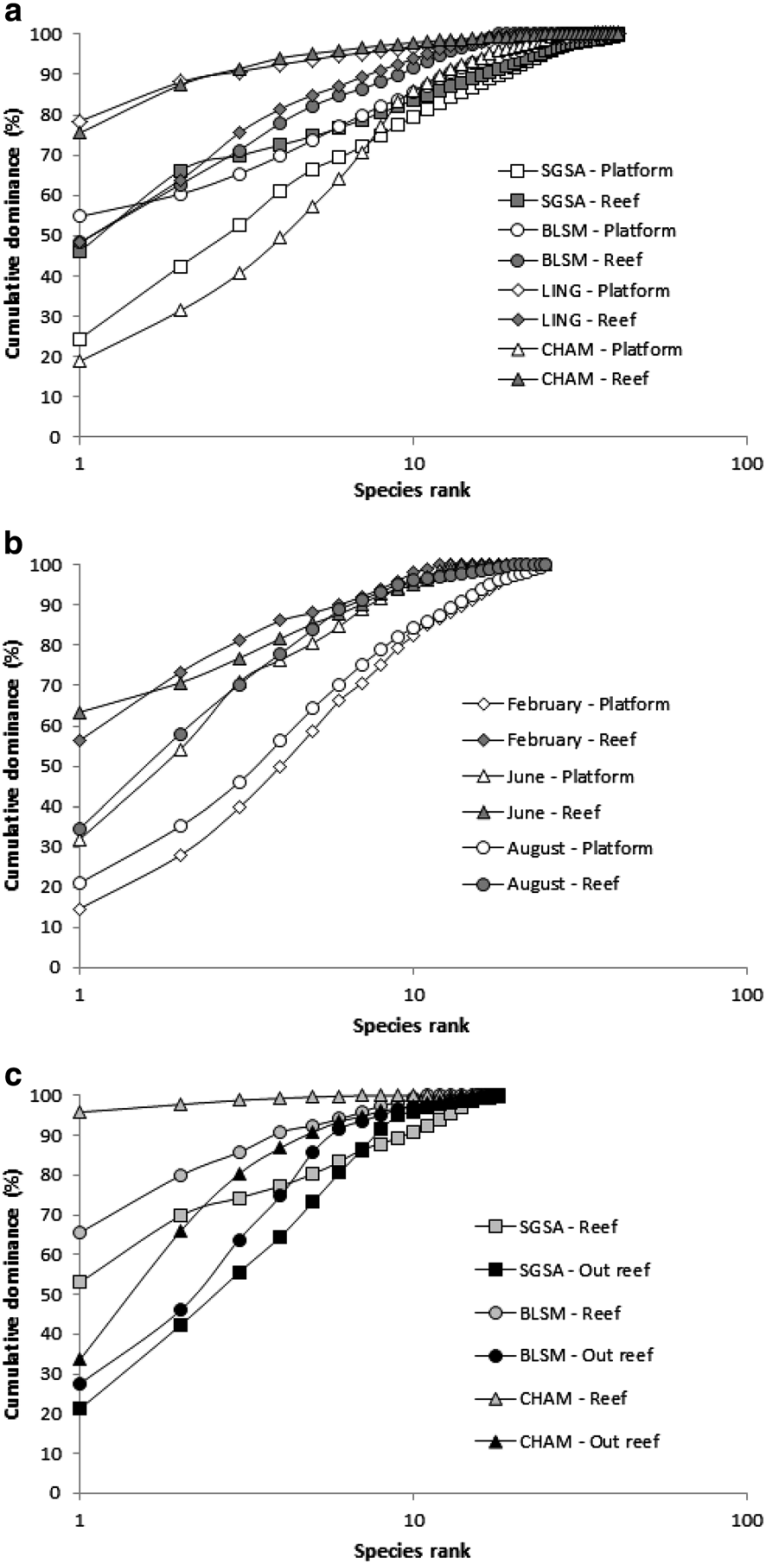
*k*-dominance curves excluding *S. alveolata*. **a** Spatial comparison (core sampling), **b** Temporal comparison for BLSM (core sampling), **c** inside and outside reefs (quadrat sampling). *CHAM* Champeaux, *LING* Lingreville, *BLSM* Blainville-sur-Mer, *SGSA* Saint-Germain-Sur-Ay

The SGSA site shows the highest TR, both on the platforms and on the reefs (Fig. 4). Conversely, BLSM has the lowest TR. Mean values of SR, N1 and N2 on the four sites are higher on platforms than on reefs, except at SGSA (Fig. 4a). Figure 5a presents *k*-dominance curves for species abundance on each site, allowing us to identify additional differences in community structure: the CHAM reef community is dominated by *S. alveolata* (75 % of total abundance) (Table 3). The LING platform community is clearly dominated by *Porcellana platycheles* (78 % of total abundance). The composition of the macrofauna on the platform structures appears more erratic between sites. Moreover, the dominance of species on platforms at SGSA and CHAM is more equally distributed than at the two other sites, with dominant species showing lower abundances.

### Temporal pattern of associated macrofauna on platform and reef structures at BLSM (core sampling)

Sampling period and structure type are significant independent factors influencing the taxonomic richness (Table 6), with a higher TR on platform structures and during August (Tukey test). As regards the total abundance, only the sampling period appears as a significant factor (Table 6), with higher abundances during August (Tukey test).

**Table 6:** Results of two-way ANOVA on taxonomic richness and total abundance values for the temporal pattern of associated macrofauna on platform and reef structures at BLSM (core sampling)

	Taxonomic richness			Total abundance		
	Df	F	*p*	Df	F	*p*
Date (A)	2	17.21	<0.01	2	15.44	<0.01
Reef/platform (B)	1	5.85	<0.05	1	0.12	0.73
A × B	2	0.72	0.49	2	0.67	0.52
∑	6			6		

F: value of the Fisher law; *p*: probability of the factor or interaction; A = samples made at three dates; B = stations sampled in two structures; (A × B) = interaction between factor A and B

**Fig. 6:**
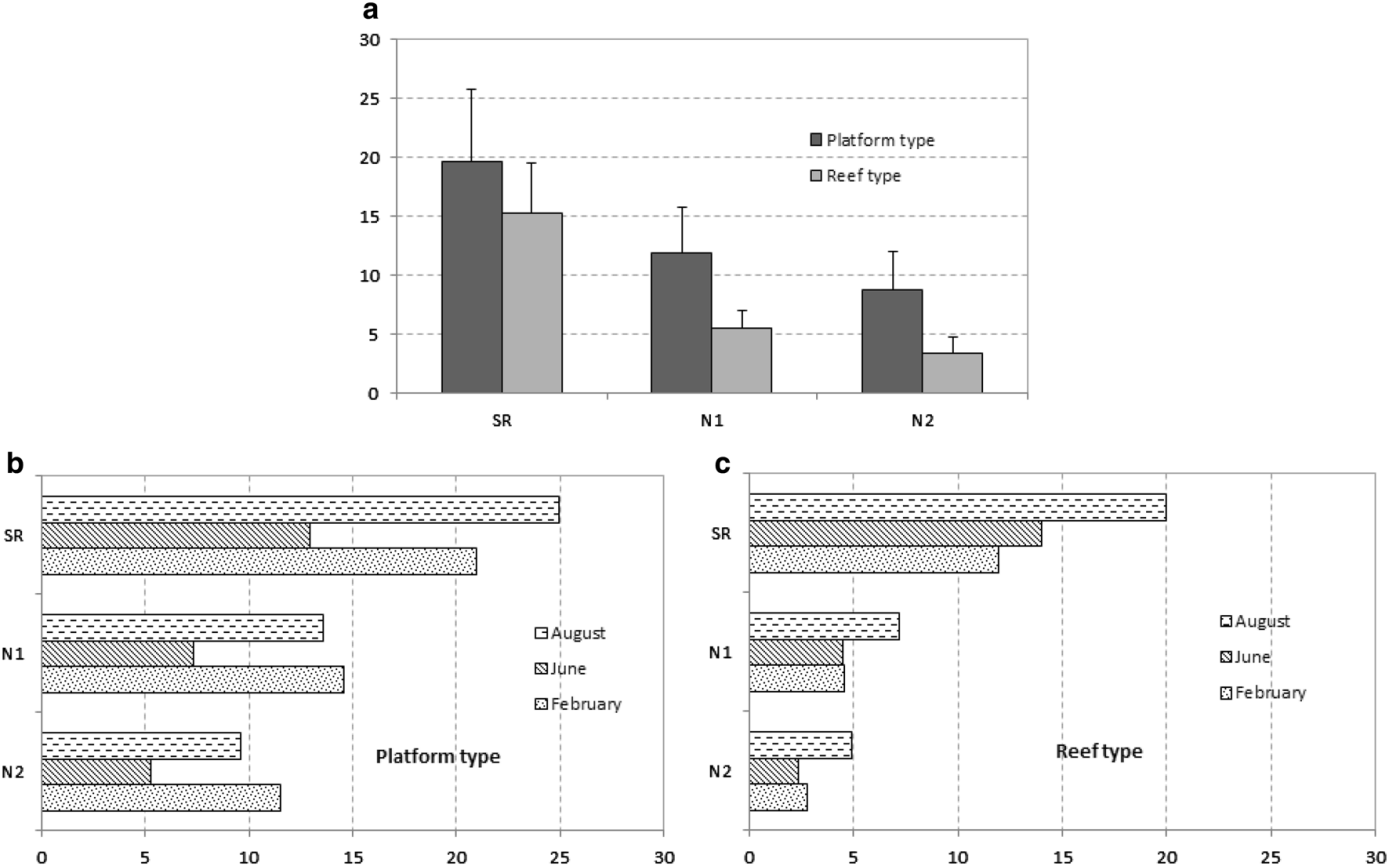
**a** Values of species richness (SR) and Hill’s numbers (N1, N2) for platform and reef-type structures (core sampling); **b, c** values of SR, N1 and N2 for platform and reef-type structures (values excluding *S. alveolata*, for different sampling dates at Blainville-sur-Mer)

In addition, we observe a change in the community structure from winter to summer (Table 4; Fig. 6). Indeed, when comparing the two substrates at the three sampling periods, the four most dominant species, excluding *S. alveolata*, are never the same. *K*-dominance curves reveal additional differences in species abundance between platform and reef (Fig. 5b). These curves show a more balanced distribution of numbers of individuals according to species for the platform than for the reef. The curve for the reef structure indicates that one or two species are dominant in February and June, while there is a more balanced distribution in August.

### Taxonomic richness and macrofauna abundance inside and outside reef structures (quadrat sampling)

**Fig. 7:**
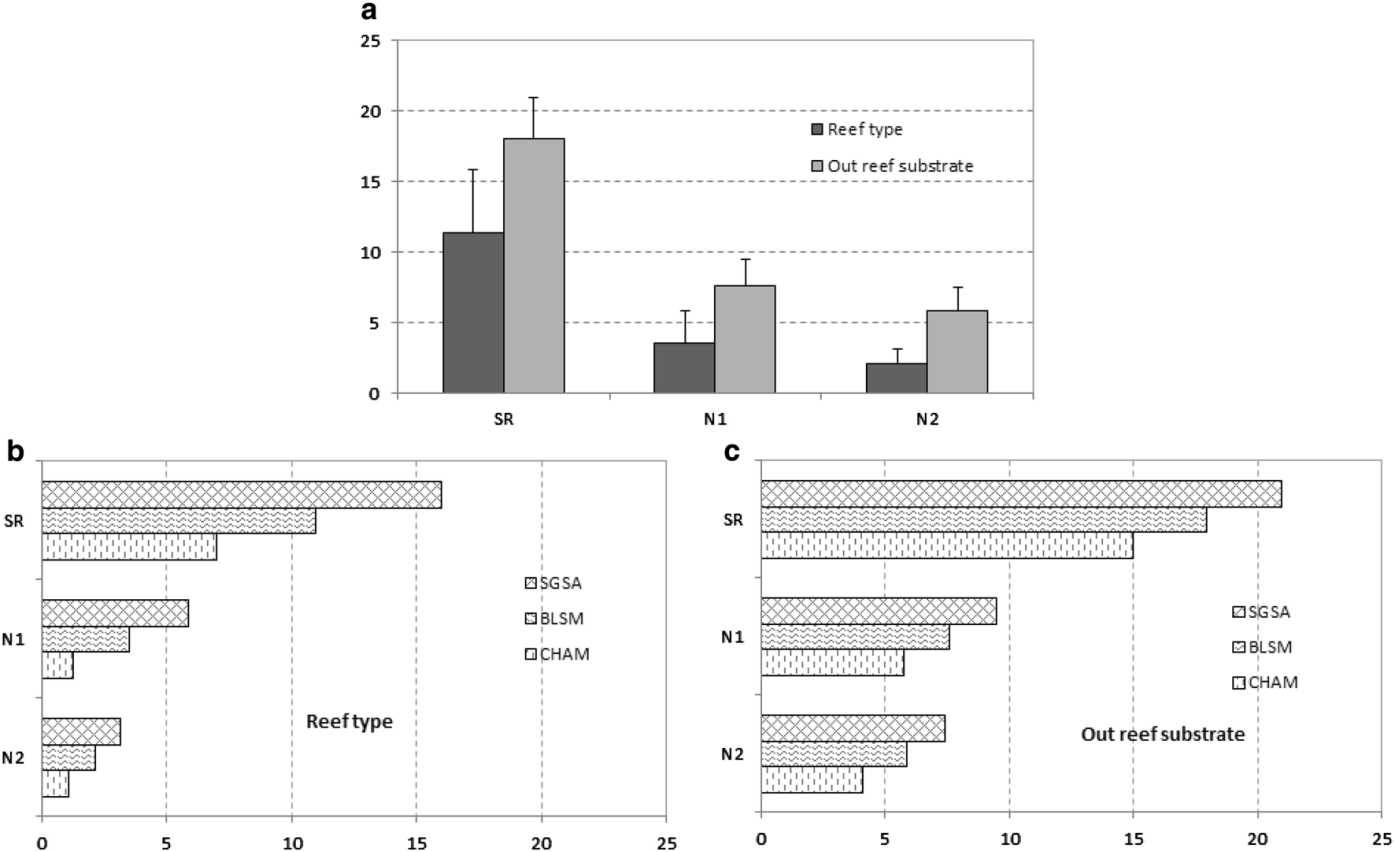
**a** Values of species richness (SR) and Hill’s numbers (N1, N2) inside and outside reefs (quadrat sampling); **b, c** values of SR, N1 and N2 inside and outside reefs (values without *S. alveolata). CHAM* Champeaux, *BLSM* Blainville-sur-Mer, *SGSA* Saint-Germain-Sur-Ay

When comparing the macrofauna inside and outside reef structures, we find that both factors (with interaction) have a significant effect on the TR and total abundance (Table 7). Because of the absence of *Sabellaria alveolata* at LING, only the SGAA and CHAM sites are included in the analyses. The mean values of SR, N1 and N2 indicate a higher TR outside than inside the reef constructions (Fig. 7a), with a total of 47 taxa (23 taxa recorded on the reef type as against 35 taxa outside). A comparison of the spatial pattern between the three sites shows that diversity indices are higher at SGSA (Fig. 7b, c), while CHAM yields the lowest values. The fauna of CHAM on substrates outside the reef is different from that recorded at the other stations, with species characteristic of soft substrates such as the bivalves *Spisula solida*, *Macoma balthica* and *Venerupis philippinarum* (Table 8; see also “Appendix 3”). The most abundant species at the other stations are represented essentially by epifauna.

**Table 7:** Results of Two-way ANOVA on taxonomic richness and total abundance values inside and outside reef structures (quadrat sampling)

	Taxonomic richness			Total abundance		
	Df	F	*p*	Df	F	*p*
Inside/outside (A)	1	28.45	<0.01	1	15.05	<0.01
Site (B)	2	7.02	<0.05	2	6.41	<0.05
A × B	2	3.96	<0.05	2	16.77	<0.01
∑	6			6		

F: value of the Fisher law; *p*: probability of the factor or interaction; A = stations sampled inside or outside the reef; B = samples made in three sites; (A × B) = interaction between factor A and B

**Table 8:** Top-ranked species densities (quadrat sampling, mean per 0.1 m^2^ ± SD) of macrofauna inside and outside *Sabellaria alveolata* reefs at three sites from the west coast of Cotentin

Site	Rank	Inside Reef		Outside Reef	
		Species	Mean density ± SD		Mean density ± SD
Saint-Germain-sur-Ay		16^a^–3.9^b^		21^a^–7.9^b^	
	1	*Sabellaria alveolata*	22.90 ± 15.62	*Gibbula umbilicalis*	11.00 ± 11.87
	2	*Gibbula umbilicalis*	3.50 ± 3.31	*Boccardia polybranchia*	10.90 ± 27.62
	3	*Gibbula pennanti*	1.10 ± 1.66	*Gibbula cineraria*	6.90 ± 6.21
	4	*Gibbula cineraria*	0.30 ± 0.67	*Patella ulyssiponensis*	4.70 ± 3.33
	5	*Mytilus edulis*	0.20 ± 0.63	*Patella vulgata*	4.50 ± 5.04
Blainville-sur-Mer		11^a^–3.7^b^		18^a^–5.3^b^	
	1	*Sabellaria alveolata*	43.40 ± 24.36	*Gibbula pennanti*	8.50 ± 11.31
	2	*Gibbula umbilicalis*	7.80 ± 7.39	*Gibbula umbilicalis*	5.70 ± 7.04
	3	*Gibbula pennanti*	1.70 ± 3.68	*Gibbula cineraria*	5.50 ± 12.34
	4	*Gibbula cineraria*	0.70 ± 2.21	*Patella ulyssiponensis*	3.40 ± 3.37
	5	*Nassarius reticulatus*	0.60 ± 0.84	*Patella vulgata*	3.40 ± 3.50
Champeaux		7^a^–3.3^b^		15^a^–4.4^b^	
	1	*Sabellaria alveolata*	99.00 ± 58.93	*Spisula solida*	6.38 ± 2.97
	2	*Sphaeroma spp*	55.20 ± 42.54	*Macoma balthica*	6.13 ± 5.57
	3	*Golfingia vulgaris*	1.10 ± 1.97	*Sphaeroma spp*	2.75 ± 3.81
	4	*Mytilus edulis*	0.60 ± 0.97	*Venerupis philippinarum*	1.25 ± 1.39
	5	*Carcinus maenas*	0.30 ± 0.48	*Crepidula fornicata*	0.75 ± 2.12

See “Appendix 3” for taxonomic details and complete species list

^a^Total species number and

^b^Mean species/number per 0.1 m^2^ quadrat

*K*-dominance curves for species abundance inside and outside reefs at each site provide additional information on the structure of the communities (Fig. 5c). Curves for the outside- reef substrate show a more balanced distribution of numbers of individuals according to species than on the reef structure. The reef community is heavily dominated by a single species, which alone accounts for 95 % of the total abundance of species at the CHAM site. The spatial distinction (reef and outside-reef) indicates that the SGSA site has a more uniform distribution of species, unlike CHAM.

## Discussion

In this study, the taxonomic richness of the macrofauna is estimated at 93 taxa (77 identified to species level) based on samples collected from four sites (“Appendix 4”). Among the recorded taxa, 13 were sampled only outside the *Sabellaria alveolata* platforms and reefs.

Accounting for 36 taxa, polychaetes dominate the taxonomic richness on *Sabellaria* platforms and reefs along the west coast of Cotentin. The total number of taxa (80) found on these *Sabellaria* bio-constructions is of the same order of magnitude as that observed by Anadon (1981) on the *Sabellaria* reefs of the Ria de Vigo in Galicia, Spain. On the Cotentin coast, 26 polychaetes are recorded, with a dominance of the Phyllodocidae (*Eumida* spp.), Nereidae (*Perenereis* spp.) and Serpulidae (*Spirobranchus triqueter).* On the Portuguese coast, the total number of taxa is 137, which appears higher than on the Cotentin coast. Polychaetes are the most abundant group on the Cotentin reefs, while the crustaceans dominate in the case of Ria de Vigo. Porras et al. (1996) determined a total of 27 polychaete taxa with a numerical dominance of Cirratulidae, Syllinae, Serpulidae, Nereidae and Phyllodocidae in the *Sabellaria* reefs of the Gulf of Valencia (Spain). On the other hand, La Porta and Nicoletti (2009) recorded a total of 39 polychaetes from the *Sabellaria alveolata* reefs of the central Tyrrhenian Sea (Italy), where the most abundant associated families are the Nereidae, the Phyllodocidae and the Serpulidae. Apart from the polychaetes, the most diversified zoological groups identified in the *Sabellaria* reefs of the western coast of Cotentin are the molluscs, including bivalves and gastropods, and the arthropods, including crustaceans (“Appendix 4”). Among the molluscs, three main categories of taxa are identified: (1) sessile epibiont species, such as the mussel *Mytilus edulis* and the oyster *Crassostrea gigas,* (2) vagile epifauna such as the gastropod *Gibbula* spp. and (3) infauna species such as *Venerupis corrugata*, the cockle *Cerastoderma edule* and the Baltic tellin *Macoma balthica* (but only at the Champeaux site). The arthropods are dominated by the decapods *Porcellana platycheles* and the isopods *Sphaeroma* spp. and *Gnathia dentata*. The sipunculid *Golfingia vulgaris* is present in abundance at the four sampled sites. The fauna collected on the *Sabellaria* bio-constructions is in common with that of the surrounding hard-bottom and soft bottom substrates, especially the muddy sand and gravel sediments which favour the presence of infauna.

Some taxonomic particularities have been highlighted by Gruet (1970, 1981, 1982), who studied the associated fauna in two areas colonized by *Sabellaria* on the coast of France (in the Bay of Bourgneuf on the Atlantic coast, and in the Bay of Mont-Saint-Michel bordering the western basin of the English Channel).

Gruet (1971) explored the fauna associated with different phases in the construction and destruction of reefs in the Bay of Bourgneuf (growth, flourishing, degradation, death and colonization) in comparison with the surrounding intertidal soft and hard substrates. In the case of dead reefs, Gruet (1971, 1972b) observed that the fauna was characteristic of the surrounding intertidal fauna, with the exception of very high densities of the amphipod *Corophium volutator*, which is a typical species of intertidal estuarine mudflats. Finally, this author (*op. cit.*) stressed that TR was higher during the colonization and destruction phases than during the flourishing reef phase. Subsequently, Gruet (1977) noted some particular features of the associated fauna of the *Sabellaria* reefs in relation to local topography and hydrodynamics.

Gruet (1970) was the first to describe in detail the associated fauna of the famous Saint-Anne reef of the Bay of Mont-Saint-Michel. He recorded a total of 60 taxa, and highlighted the abundance of crevice and cavity species such as the polychaete *Eulalia viridi*s, the isopod *Spaeroma monodi* and the sipunculid *Golfingia vulgaris*. These three taxa are among the most dominant in our samples (Tables 3, 4, 8).

Dubois et al. (2002) described the macrofauna associated with the Saint-Anne reef, showing that polychaetes dominate the fauna (41 taxa), and that the associated fauna is more diverse than in the soft-bottom surrounding habitats; the total number of taxa counted in March 2000 from 24 samples was 63, which is lower than in our study (80). However, Dubois et al. (2002) collected a larger number of samples (124) using replicate quadrats ranging in area from 0.032 to 0.1 m^2^. TR was found to change within the three stages of reef evolution, i.e. Ball-shaped structures, Platform and Degraded reef. These findings are similar to the results obtained in the present study, which reveal differences in fauna between the platform, reef and degraded reef at Lingreville. Hence, TR appears to be a function of the heterogeneity of the substrate and the successive stages. Moreover, our quadrat samples show that TR is higher outside the reef structure than inside: (1) the fauna recorded on the reef is in common with the surrounding hard and soft-bottom substrates; (2) the TR decreases with increasing abundance of the engineer species *Sabellaria alveolata*, which shows very dense populations >20,000–60,000 ind m^−2^.

More recently, Dubois et al. (2006) studied the effect of the presence of epibionts (e.g. green algae (*Ulva* spp.) and the oyster *Crassostrea gigas*) on the TR of the Saint-Anne reef. These authors showed that the presence of the oyster had a significant positive effect on TR compared with areas lacking *C. gigas* and with algal-reef type structures.

Moreover, Gruet (1981) has provided some information on the fauna associated with living and dead *Sabellaria* platforms at Champeaux. This author (op.cit.) stressed that the fauna was poor in terms of TR (26 taxa identified) and abundance, probably in relation to its location on the upper part of the mid-littoral zone with a long period of exposure of the *Sabellaria* reef. We identify 38 taxa in our samples from the Champeaux site, but our results are based on a more extensive sampling campaign.

Nevertheless, on the west coast of Cotentin, the TR appears to vary between different sites and structures, being higher on the platforms than on the reefs, and higher outside than inside the reef (Tables 3, 4, 8; Figs. 4, 6). This could be due to the fact that *S. alveolata* has developed as a flourishing reef monopolizing the area and volume by imposing a strong competition with other species. In this way, *S. alveolata* has occupied all the available space, where its density reaches and can exceed several thousands of individuals per square metre (Gruet 1971, 1982, 1986). Moreover, Porras et al. (1996), Dubois et al. (2002), and Desroy et al. (2011) pointed out that, in degraded reefs, including eroded reefs during the destruction phase, the TR is much higher than during the other phases of reef evolution. Well-developed reefs show the lowest levels of polychaete TR at several locations such as in Galicia, Spain (Anadon 1981), in the Valencia Gulf (Porras et al. 1996) and in the Tyrrhenian Sea (La Porta and Nicoletti 2009). High TR could be explained by fragmentation of the bio-constructions (Gruet 1970, 1971, 1972a, b, 1977, 1981, 1982, 1986) which leads to a greater structural complexity (Porras et al. 1996). The heterogeneity of the substrate due to the presence of dead or living reef structures gives it an irregular nature with few crevices, which favours the colonization of *Sabellaria* bio-constructions by species from soft- and hard-bottom surrounding habitats. Indeed, during the growth of reef structures, slumps and cracks are formed which create numerous more or less concealed cavities providing shelter for many animals (Gruet 1982). Moreover, since the structures do not evolve synchronously—as in the case of the Champeaux reef—this leads to the persistence of reefs in this area. Because of this, the associated benthic communities always find a favourable place to settle and the reef becomes colonized by species from the surrounding hard-bottom and soft-bottom habitats.

Furthermore, as highlighted by Dubois et al. (2006) the *S. alveolata* bio-constructions show a high TR since the faunal assemblages are composed of associations of taxa typically found on various hard and soft-bottom substrates. Thus, the TR within bio-constructions gives rise to a wide variety of trophic patterns making up a food chain involving not only suspension feeders and detritivores, but also scavengers (Gruet 1982). On the Lingreville site, we note the absence of *S. alveolata* associated with high abundances of *Porcellana platycheles*. According to Gruet (1982), *P. platycheles*, like *Carcinus maenas*, would be one of the main predators of *S. alveolata*.

The environmental conditions associated with a given bio-construction also play an important role in controlling diversity and abundance. In fact, one of the factors producing faunal changes is the position of the structure in the intertidal zone, between the upper and lower part of the mid-littoral zone (Gruet 1982). Indeed, at the four sites studied here, bio-constructions are not all at the same elevation with respect to sea level. The structures at Champeaux are developed in the upper part of the mid-littoral zone, at the foot of a 20-m-high cliff, while the structures of Lingreville, Blainville-sur-Mer and Saint-Germain-sur-Ay are located on the middle part of the mid-littoral zone. In addition, the exposure of the coast to hydrodynamic action, including tidal currents, swell and waves, also plays a major role in controlling the TR found within bio-constructions. Indeed, Gruet (1971 and 1982) observed higher values of TR in the bays, while exposed areas were less favourable for the development of high TR. As regards the present study, Champeaux is located in a more sheltered area compared with open sites exposed to higher energy hydrodynamic conditions such as Lingreville, Blainville-sur-Mer and Saint-Germain-sur-Ay.

The age of the biogenic formation also influences the TR developed on a given structure: this ranges from 5 to 8 years on the western coast of Cotentin. Indeed, Gruet (1971, 1982) pointed out that a certain lapse of time is required for a benthic community to settle, grow and (theoretically) reach equilibrium or a certain degree of maturity. In this context, the TR would be initially low, reaching a stable value indicating equilibrium of the community, while the highest TR would then be recorded along reefs. The time-evolution of TR would appear to be mainly due to the heterogeneity of the habitat and the age of the reef construction (minimum of 3–5 years).

During the 2014 sampling campaign, the surface-area of the platform and reef formations was of the same order magnitude as in 2010–2011: 2.28 km^2^ in 2010–2011 as against 2.48 km^2^ in 2014 (Basuyaux 2011; Lecornu 2014; Lecornu et al. 2016). At the scale of the four studied sites, the surface-area has remained stable at SGSA (0.11 km^2^ in 2010–2011, as against 0.12 km^2^ in 2014), but is increasing at CHAM (from 0.31 to 0.37 km^2^). There has been a marked decrease in the area covered by bio-constructions at the two other sites (falling from 0.80 to 0.61 km^2^ at BLSM and from 0.18 to 0.10 km^2^ at LING). At these two latter sites, there has also been an increase in the area covered by degraded reef. Destruction has been observed at BLSM since 2011 (Lecornu 2014; Lecornu et al. 2016) and is continuing up to the present (Dauvin, personal observation): the disappearance of reef-type and degraded platform bio-constructions is probably due to high sedimentation rates, with high-energy hydrodynamics favouring the transport of sediment and *Ruditapes* clams into the same area (Beck et al. 2015). This decline in the *Sabellaria* population was observed in the 2014 survey of BLSM, so the decrease in abundance of the honeycomb worm reflects a rapid local change between the middle of the winter and the summer, during a destruction phase, followed by colonization by algae (Table 4 and “Appendix 2”).

Moreover, recreational fishing for shrimps, crabs and other target species is concentrated on the low mid-littoral and infralittoral fringe on the west coast of Cotentin, whereas *Sabellaria* bio-constructions are located on the middle and upper mid-littoral zones. Reef deterioration caused by human activities is very limited on this part of the coastline and does not represent the same challenge for preservation of the natural heritage as the reefs in the Bay of Mont-Saint-Michel (Desroy et al. 2011). The degradation of the reefs is mainly due to natural factors, such as the hydrodynamic regime and sediment transport in an area with strong tidal currents, and the frequent occurrence of severe winter storms along the Atlantic and English Channel coastlines since the beginning of the 2010 s, especially in 2014. These extreme events could be the consequence of climate changes linked to the anthropogenic increase in atmospheric carbon dioxide.

The importance of *Sabellaria alveolata* (Linnaeus 1767) (Polychaeta: Sabellariidae) reefs has led to their classification as a remarkable natural habitat (listed in Annex I of the Habitats Directive 92/43/EEC; Natura 2000). Thus, *Sabellaria alveolata* reefs are characterized by their great importance for the conservation of natural heritage and marine biodiversity in Europe. The French national inventory of natural heritage (INPN) has recognized *Sabellaria alveolata* reefs (Habitat code 1170-4; Bensettiti et al. 2004) as representing a highly original and localized habitat with high diversity, including rare species. Bensettiti et al. (2004) stresses that a maximal protection of reefs is desired to ensure their sustainability. These authors suggest that the monitoring of water quality is essential for the conservation of these reefs and that human trampling is to be avoided.

The Bay of Mont-Saint-Michel is classified as a Special Zone of Conservation and is a RAMSAR zone (https://inpn.mnhn.fr/docs/natura2000/fsdpdf/FR2500077.pdf). Consequently, the Champeaux and the Sainte-Anne reefs are included in this Marine Protected Area. Monitoring studies have been carried out on these reefs for two decades (Dubois et al. 2002, 2006; Desroy et al. 2011). Farther north, there is no specific MPA and no management measures have been implemented concerning honeycomb worm reefs. In this area, there is a project to set up a Natural Marine Park (http://www.aires-marines.fr/L-Agence/Organisation/Missions-d-etude-de-parc/Golfe-normand-breton), which will involve the mapping and management of natural habitats including *S. alveolata* reefs. In this process, the presence of temporary *S. alveolata* reefs merits special attention.

## Conclusions

The objective of this study is to estimate the macrofaunal TR associated with *Sabellaria* bio-constructions (platform and reef types) on hard substrates along the west coast of the Cotentin Peninsula. Spatial analysis allows us to distinguish three groups among the studied sites. Champeaux is different from the other sites because the reef has developed on a soft substrate and the platform is located in the upper part of the mid-littoral zone. Lingreville is characterized by the presence of dead reef, which clearly reflects a degraded phase. The sites of Blainville-sur-Mer and Saint-Germain-sur-Ay are located in the north of the study area, and show similar patterns. The different study sites are found to display highly variable states ranging from a flourishing reef at Champeaux to a completely degraded reef at Lingreville. Some reef destruction is also observed at Blainville-sur-Mer. These results highlight a difference in TR between platforms and reefs, with platforms showing higher TR. Such variability supports the reef effect proposed in previous studies, i.e. a decrease in the TR of other resident macrofauna species in zones more intensely colonized by the honeycomb worm *Sabellaria alveolata*. Therefore, well-developed reefs in a flourishing state do not represent the most diversified habitats in terms of TR. This is because *S. alveolata* occupies all the space, inhibiting the creation of crevices, fissures and pits which offer the best refuges for TR in mosaic habitats developed during the construction and destruction phases of a reef. The project for the creation of a Natural Marine Park should be an opportunity to recognize and manage the natural heritage interest of such temporary reefs on hard bottom habitats along the western coast of Cotentin.

## Appendix 1

See Table 9.

**Table 9:** Species abundances (mean per 1/32 m^2^ ± SD) of macrofauna associated with *Sabellaria alveolata* structures

Taxa	Platform type				Reef type			
	CHAM	LING	BLSM	SGSA	CHAM	LING	BLSM	SGSA
*Sabellaria alveolata*	1182.88 ± 676.30	1.50 ± 2.07	130 ± 53.86	80.00 ± 31.21	771.88 ± 265.52	0.15 ± 0.35	115.75 ± 140.50	242.75 ± 77.12
Polychaete—35 taxa								
*Amphitritides gracilis*	–	–	0.13 ± 0.35	0.38 ± 1.06	–	–	–	–
*Aonides oxycephala*	–	–	–	0.50 ± 1.07	–	–	–	0.25 ± 0.71
*Arabella iricolor*	–	–	–	–		0.13 ± 0.35	–	–
*Branchiomma lucullanum*	–	–	0.13 ± 0.35	0.63 ± 1.19	–	–	0.13 ± 0.35	0.63 ± 1.41
*Capitella* sp	2.38 ± 4.47 **[5]**	–	–	–		–	–	–
*Caulleriella* sp	–	–	–	0.13 ± 0.35	–	–	–	–
*Cirratulus cirratus*	0.75 ± 1.16	0.13 ± 0.35	–	–	–	–	–	2.63 ± 7.42 **[3]**
*Eteone longa*	0.13 ± 0.35	–	–	–	–	–	–	–
*Eulalia ornata*	5.63 ± 4.27 **[1]**	–	0.38 ± 0.52	–	0.13 ± 0.35	–	–	–
*Eumida sanguinea*	1.88 ± 1.36	0.63 ± 0.92	–	0.63 ± 1.06	0.75 ± 0.89	0.38 ± 0.74	0.25 ± 0.71	0.75 ± 1.04
*Glycera tridactyla*	0.25 ± 0.46	0.13 ± 0.35	–	–	0.13 ± 0.35	–	–	–
*Goniadella bobrezkii*	0.38 ± 0.74	0.13 ± 0.35	–	–	–	–	–	–
Hesionidae	–	0.25 ± 0.71	–	–	–	–	–	–
*Hilbigneris gracilis*	–	–	0.50 ± 0.53	1.38 ± 2.13	–	–	0.13 ± 0.35	0.13 ± 0.35
*Lanice conchilega*	–	–	–	3.50 ± 2.98 **[5]**	–	–	–	0.13 ± 0.35
*Lysidice ninetta*	–	–	–	–	–	–	–	0.13 ± 0.35
*Lysidice unicornis*	–	–	–	–	–	–	–	0.13 ± 0.35
*Malacoceros fuliginosus*	2.75 ± 4.30 **[3]**	1.75 ± 2.19 **[5]**	0.13 ± 0.35	0.63 ± 1.119	–	–	–	0.38 ± 1.06
Maldanidae	–	–	0.13 ± 0.35	0.13 ± 0.35	–	–	–	0.13 ± 0.35
*Malmgreniella ljungmani*	–	–	0.13 ± 0.35	0.25 ± 0.46	–	–	–	–
*Marphysa sanguinea*	–	–	0.13 ± 0.35	–	–	–	–	–
*Nereis caudata*	–	–	–	0.13 ± 0.35	–	–	–	–
*Notomastus latericeus*	0.13 ± 0.35	0.13 ± 0.35	0.13 ± 0.35	6.63 ± 6.30 **[3]**	–	–	–	0.50 ± 0.76
Paraonidae	–	–	0.13 ± 0.35	–	–	–	–	–
*Perinereis cultrifera*	3.75 ± 4.03 **[2]**	0.25 ± 0.46	1.13 ± 1.81 **[3]**	1.00 ± 1.41	0.13 ± 0.35	0.63 ± 0.52	1.00 ± 1.41 **[4]**	1.50 ± 1.51 **[5]**
*Pholoe inornata*	0.13 ± 0.35	–	–	–	–	–	–	–
*Phyllodoce laminosa*	–	–	–	–	0.13 ± 0.35	–	–	0.13 ± 0.35
*Phyllodoce mucosa*	0.25 ± 0.46	0.25 ± 0.46	–	–	–	–	–	–
*Platynereis dumerilii*	–	–	0.88 ± 0.99 **[5]**	1.63 ± 1.60	–	–	–	0.38 ± 0.52
*Polycirrus medusa*	0.13 ± 0.35	–	0.38 ± 0.74	1.25 ± 2.38	–	–	–	0.50 ± 1.07
*Schistomeringos neglecta*	–	0.13 ± 0.35	–	–	–	–	–	–
*Spio* sp	0.63 ± 0.92	–	–	0.38 ± 0.74	–	0.13 ± 0.35	–	0.13 ± 0.35
*Spirobranchus triqueter*	0.25 ± 0.71	–	1.00 ± 1.07 **[4]**	1.88 ± 2.42	0.50 ± 0.76	–	–	1.13 ± 1.13
*Sthenelais boa*	–	–	–	0.13 ± 0.35	–	–	–	–
Syllidae	2.00 ± 2.00	0.13 ± 0.35	0.25 ± 0.46	0.38 ± 0.74	0.25 ± 0.46	–	–	0.63 ± 1.19
Mollusca—18 taxa								
*Acanthochitona crinita*	–	–	1.25 ± 1.39 **[2]**	0.50 ± 0.53	–	–	0.13 ± 0.35	0.50 ± 0.53
*Cerastoderma edule*	0.13 ± 0.35	–	–	–	–	–	–	–
*Crassostrea gigas*	–	–	0.13 ± 0.35	–	–	–	–	–
*Crepidula fornicata*	–	0.13 ± 0.35	–	–	–	–	–	–
*Gibbula cineraria*	–	0.13 ± 0.35	–	0.50 ± 0.76	–	–	–	0.13 ± 0.35
*Gibbula pennanti*	–	1.00 ± 1.31	–	0.63 ± 1.19	–	–	0.38 ± 0.74	0.75 ± 1.16
*Gibbula umbilicalis*	0.13 ± 0.35	2.25 ± 2.82 **[4]**	0.25 ± 0.71	5.75 ± 3.41 **[4]**	–	3.00 ± 1.85 **[4]**	7.13 ± 7.85 **[1]**	1.38 ± 1.77
*Goodallia triangularis*	–	0.13 ± 0.35	–	–	–	0.13 ± 0.35	–	–
*Littorina littorea*	–	–	–	–	–	–	0.25 ± 0.71	–
*Littorina obtusata*	–	–	–	–	–	–	0.25 ± 0.46	–
*Mytilus edulis*	0.63 ± 0.74	3.13 ± 2.47 **[3]**	–	0.63 ± 1.06	0.50 ± 1.41	1.13 ± 1.25	0.25 ± 0.46	0.88 ± 1.46
*Nassarius reticulatus*	–	0.25 ± 0.46	–	0.38 ± 1.06	–	–	–	0.63 ± 1.06
*Nucella lapillus*	–	0.25 ± 0.71	–	–	–	–	–	–
*Ocenebra erinaceus*	0.13 ± 0.35	–	–	–	0.25 ± 0.46	–	–	–
*Ruditapes philippinarum*	0.13 ± 0.35	–	0.13 ± 0.35	–	0.13 ± 0.35	–	–	–
*Spisula solida*	–	–	–	–	0.38 ± 0.74	–	–	–
*Trivia monacha*	–	–	–	–	–	–	–	0.13 ± 0.35
*Venerupis corrugata*	0.13 ± 0.35	0.25 ± 0.46	0.75 ± 1.04	0.75 ± 0.46	0.25 ± 0.46	0.38 ± 0.52	0.63 ± 0.52 **[5]**	0.63 ± 0.74
Arthropoda—18 taxa								
*Ampithoe rubricata*	–	0.25 ± 0.71	0.13 ± 0.35	0.88 ± 1.81	–	0.25 ± 0.71	–	0.13 ± 0.35
*Cancer pagurus*	–	–	–	–	0.25 ± 0.71	–	–	0.13 ± 0.35
*Carcinus maenas*	0.38 ± 0.74	0.75 ± 0.71	0.13 ± 0.35	0.88 ± 0.83	1.00 ± 1.07	0.25 ± 0.46	0.13 ± 0.35	1.13 ± 0.83
*Cheirocratus* spp	–	0.25 ± 0.46	–	–	–	–	–	0.13 ± 0.35
*Cyathura carinata*	–	–	–	0.13 ± 0.35	–	1.13 ± 1.13	–	–
*Dynamene bidentata*	–	–	–	0.38 ± 0.52	–	–	0.25 ± 0.46	0.25 ± 0.46
*Gnathia dentata*	–	–	0.13 ± 0.35	–	0.13 ± 0.35	0.63 ± 1.41	–	–
*Idotea granulosa*	–	0.13 ± 0.35	–	–	–	–	–	–
*Melita palmata*	–	1.13 ± 0.64	–	0.13 ± 0.35	0.25 ± 0.46	0.88 ± 0.99	–	1.50 ± 1.41
*Monocorophium acherusicum*	–	0.88 ± 0.35	–	–	–	0.63 ± 0.52	–	0.25 ± 0.46
*Nymphon* sp	–	–	–	–	–	–	–	0.38 ± 0.74
*Orchomene humilis*	–	0.13 ± 0.35	–	–	–	–	–	–
*Palaemon elegans*	–	0.25 ± 0.71	–	–	–	–	–	–
*Pilumnus hirtellus*	–	–	0.38 ± 0.52	1.88 ± 2.03	0.13 ± 0.35	–	0.25 ± 0.71	1.25 ± 1.75
*Porcellana platycheles*	–	17.25 ± 26.72 **[1]**	0.63 ± 0.92	11.88 ± 14.79 **[2]**	15.75 ± 37.17 **[2]**	7.88 ± 13.25 **[2]**	2.13 ± 2.23 **[2]**	32.50 ± 30.16 **[1]**
*Sphaeroma serratum/monodi*	2.63 ± 5.07 **[4]**	140.5 ± 55.63	0.38 ± 0.74	1.88 ± 1.64	100.13 ± 66.25 **[1]**	24.38 ± 10.32 **[1]**	0.13 ± 0.35	0.63 ± 1.41
*Vaunthompsonia cristata*	0.13 ± 0.35	–	–	–	–	0.13 ± 0.35	–	–
Chordata—2 taxa								
Ascidiidae	–	–	–	0.25 ± 0.71	0.88 ± 2.47	–	–	0.38 ± 1.06
Blenniidae	–	0.25 ± 0.71	–	–	–	0.13 ± 0.35	–	0.25 ± 0.46
Sipuncula—1 taxon								
*Golfingia (Golfingia) vulgaris*	0.25 ± 0.46	0.63 ± 1.06	12.25 ± 8.41 **[1]**	16.13 ± 6.77 **[1]**	5.38 ± 9.53 **[3]**	1.75 ± 2.38 **[5]**	1.25 ± 1.49 **[3]**	14.13 ± 11.57 **[2]**
Nemertea—1 taxon								
Nemertea	1.88 ± 4.55	4.75 ± 3.54 **[2]**	0.38 ± 0.52	0.75 ± 1.04	1.50 ± 2.78 **[5]**	5.88 ± 13.04 **[3]**	0.13 ± 0.35	1.88 ± 1.73 **[4]**
Bryozoa—1 taxon								
*Electra pilosa*	–	0.13 ± 0.35	–	–	–	–	–	–
Cnidaria—1 taxon								
Actiniaria	2.00 ± 2.07	0.38 ± 0.52	–	–	3.50 ± 5.40 **[4]**	0.75 ± 1.16	–	0.13 ± 0.35
Echinodermata—1 taxon								
*Amphipholis squamata*	–	0.13 ± 0.35	–	0.13 ± 0.35	–	0.13 ± 0.35	–	0.38 ± 0.74
Insecta—1 taxon								
*Axelsonia littoralis*	–	–	–	–	0.13 ± 0.35	–	–	–

Bold numbers in brackets are ranks of the top five species (excluding *S. alveolata*)

*CHAM* Champeaux, *LING* Lingreville, *BLSM* Blainville-Sur-Mer, *SGSA* Saint-Germain-Sur-Ay

## Appendix 2

See Table 10.

**Table 10:** Species abundances (mean per 1/32 m^2^ ± SD) of macrofauna associated with *Sabellaria alveolata* on each date at Blainville-sur-Mer

Taxa	Platform type			Reef type		
	February	June	August	February	June	August
*Sabellaria alveolata*	130 ± 53.86 **[1]**	51.63 ± 44.42 **[1]**	84.38 ± 54.22 **[1]**	115.75 ± 140.50 **[1]**	42.63 ± 38.63 **[1]**	16 ± 16.09 **[1]**
Polychaete—16 taxa						
*Amphitritides gracilis*	0.13 ± 0.35	–	0.38 ± 0.52	–	–	–
*Aonides oxycephala*	–	–	0.38 ± 0.74	–	–	–
*Eulalia ornata*	0.38 ± 0.52	–	0.5 ± 1.07	–	–	0.25 ± 0.71
*Eumida sanguinea*	–	0.5 ± 1.07 **[5]**	–	0.25 ± 0.71	0.5 ± 0.53 **[5]**	0.13 ± 0.35
*Lanice conchilega*	–	0.38 ± 0.74	1.88 ± 1.73	–	0.25 ± 0.71	–
*Lysidice ninetta*	–	–	0.13 ± 0.35	–	–	–
Malacoceros fuliginosus	0.13 ± 0.35	–	–	–	–	–
Maldanidae	0.13 ± 0.35	–	0.25 ± 0.46	–	–	–
*Marphysa sanguinea*	0.13 ± 0.35	–	–	–	–	–
*Nereis caudata*	–	0.13 ± 0.35	–	–	0.25 ± 0.46	–
*Notomastus latericeus*	0.13 ± 0.35	–	2.38 ± 1.69 **[5]**	–	–	–
*Perinereis cultrifera*	1.13 ± 1.81 **[3]**	–	0.88 ± 1.25	1 ± 1.41 **[4]**	–	2.75 ± 1.49 **[5]**
*Platynereis dumerilii*	0.88 ± 0.99 **[5]**	–	3.25 ± 4.89 **[3]**	–	–	0.38 ± 0.52
*Polycirrus medusa*	0.38 ± 0.74	–	–	–	–	–
*Spirobranchus triqueter*	1 ± 1.07 **[4]**	0.25 ± 0.71	0.38 ± 0.52	–	0.13 ± 0.35	–
Syllidae	0.25 ± 0.46	–	0.38 ± 0.74	–	–	–
Mollusca—11 taxa						
*Acanthochitona crinita*	1.25 ± 1.39 **[2]**	0.38 ± 0.74	1.13 ± 1.13	0.13 ± 0.35	0.38 ± 0.74	–
*Crassostrea gigas*	0.13 ± 0.35	–	–	–	–	–
*Gibbula cineraria*	–	–	–	–	0.13 ± 0.35	–
*Gibbula umbilicalis*	0.25 ± 0.71	2 ± 1.60 **[3]**	4.88 ± 4.36 **[2]**	7.13 ± 7.85 **[2]**	6.5 ± 3.66 **[2]**	12.38 ± 12.83 **[2]**
*Littorina littorea*	–	–	0.13 ± 0.35	0.25 ± 0.71	0.13 ± 0.35	0.75 ± 1.39
*Littorina obtusata*	–	–	–	0.25 ± 0.46	–	–
*Mytilus edulis*	–	–	0.13 ± 0.35	0.25 ± 0.46	0.13 ± 0.35	0.13 ± 0.35
*Nassarius reticulatus*	–	–	0.13 ± 0.35	–	–	–
*Nucula hanleyi*	–	–	0.38 ± 0.74	–	–	–
*Ruditapes philippinarum*	0.13 ± 0.35	–	–	–	–	0.13 ± 0.35
*Venerupis corrugata*	0.75 ± 1.04	0.38 ± 0.74	0.75 ± 0.71	0.63 ± 0.52 **[5]**	0.75 ± 1.03 **[3]**	0.13 ± 0.35
Arthropoda—11 taxa						
*Ampithoe rubricata*	0.13 ± 0.35	–	–	–	–	0.63 ± 1.41
*Cancer pagurus*	–	0.13 ± 0.35	–	–	–	0.13 ± 0.35
*Carcinus maenas*	0.13 ± 0.35	–	2.5 ± 2.51 **[4]**	0.13 ± 0.35	0.13 ± 0.35	1.75 ± 2.05
*Cyathura carinata*	–	0.25 ± 0.71	0.38 ± 1.06	–	–	8.38 ± 6.16 **[3]**
*Dynamene bidentata*	–	1.5 ± 1.60 **[4]**	0.25 ± 0.46	0.25 ± 0.46	0.25 ± 0.71	0.88 ± 1.25
*Gnathia dentata*	0.13 ± 0.35	0.13 ± 0.35	–	–	–	0.13 ± 0.35
*Idotea granulosa*	–	–	0.13 ± 0.35	–	–	–
*Melita palmata*	–	–	–	–	–	0.13 ± 0.35
*Monocorophium acherusicum*	–	–	–	–	–	0.13 ± 0.35
*Pilumnus hirtellus*	0.38 ± 0.52	–	–	0.25 ± 0.71	–	0.13 ± 0.35
*Porcellana platycheles*	0.63 ± 0.92	2.88 ± 5.36 **[2]**	1.38 ± 2.33	2.13 ± 2.23 **[3]**	0.63 ± 1.06 **[4]**	4.38 ± 3.25 **[4]**
Cnidaria—1 taxon						
Actiniaria	–	–	–	–	0.13 ± 0.35	2.13 ± 3.23
Echinodermata—1 taxon						
*Amphipholis squamata*	–	0.13 ± 0.35	0.13 ± 0.35	–	–	–
Insecta—1 taxon						
*Axelsonia littoralis*	–	–	0.13 ± 0.35	–	–	–

Bold numbers in brackets are ranks of top five species in each studied area

## Appendix 3

See Table 11.

**Table 11:** Species abundances (mean 0.1 m^2^ ± SD) of macrofauna associated with *Sabellaria alveolata* reef and outside reef

Taxa	Inside Reef			Outside Reef		
	CHAM	BLSM	SGSA	CHAM	BLSM	SGSA
*Sabellaria alveolata*	99.00 ± 58.93 **[1]**	43.40 ± 24.36 **[1]**	22.90 ± 15.62 **[1]**	–	–	–
Mollusca—21 taxa						
*Acanthochitona crinita*	–	0.20 ± 0.63	0.10 ± 0.32	–	0.10 ± 0.32	0.20 ± 0.63
*Crepidula fornicata*	–	–	–	0.75 ± 2.12 **[5]**	–	–
*Gibbula cineraria*	–	0.70 ± 2.21 **[4]**	0.30 ± 0.67 **[4]**	–	5.50 ± 12.34 **[3]**	6.90 ± 6.21 **[3]**
*Gibbula pennanti*	–	1.70 ± 3.68 **[3]**	1.10 ± 1.66 **[3]**	–	8.50 ± 11.31 **[1]**	3.00 ± 2.91
*Gibbula umbilicalis*	–	7.80 ± 7.39 **[2]**	3.50 ± 3.31 **[2]**	–	5.70 ± 7.04 **[2]**	11.00 ± 11.87 **[1]**
*Glycymeris*	–	–	–	0.50 ± 1.07	–	–
*Lepidochitona cinerea*	–	–	–	–	0.10 ± 0.32	–
*Littorina littorea*	–	0.20 ± 0.42	–	–	0.30 ± 0.95	2.70 ± 3.16
*Littorina obtusata*	–	–	–	–	0.40 ± 0.97	0.50 ± 0.97
*Macoma balthica*	–	–	–	6.13 ± 5.57 **[2]**	–	–
*Mytilus edulis*	0.60 ± 0.97 **[4]**	–	0.20 ± 0.63	–	–	–
*Nassarius reticulatus*	–	0.60 ± 0.84 **[5]**	–	0.13 ± 0.35	0.60 ± 0.84	0.30 ± 0.67
*Nucella lapillus*	–	–	–	–	0.50 ± 0.85	3.80 ± 4.80
*Nucula hanleyi*	–	–	–	–	0.20 ± 0.42	–
*Ocenebra erinaceus*	–	–	–	–	0.10 ± 0.32	–
*Patella intermedia*	–	–	–	–	1.80 ± 3.49	1.80 ± 1.99
*Patella ulyssiponensis*	–	–	–	–	3.40 ± 3.37 **[5]**	4.70 ± 3.33 **[4]**
*Patella vulgata*	–	–	–	–	3.40 ± 3.50 **[4]**	4.50 ± 5.04 **[5]**
*Spisula solida*	–	–	–	6.38 ± 2.97 **[1]**	–	–
*Venerupis decussata*	–	–	–	0.13 ± 0.35	0.10 ± 0.32	–
*Venerupis philippinarum*	–	–	0.10 ± 0.32	1.25 ± 1.39 **[4]**	–	–
Polychaeta—12 taxa						
*Boccardia polybranchia*	–	–	–	–	–	10.90 ± 27.62 **[2]**
*Cirratulus cirratus*	–	–	–	0.25 ± 0.71	–	–
*Eumida sanguinea*	–	–	–	–	–	0.10 ± 0.32
*Glycera tridactyla*	–	–	–	0.25 ± 0.71	–	0.10 ± 0.32
*Malacoceros fulginosus*	–	–	–	–	–	0.10 ± 0.32
*Nephtys cirrosa*	–	–	–	0.13 ± 0.35	–	–
*Nephtys hombergii*	–	–	–	0.13 ± 0.35	–	–
*Notomastus latericeus*	–	–	–	0.13 ± 0.35	–	–
*Perinereis cultrifera*	–	0.10 ± 0.32	0.10 ± 0.32	–	–	–
*Platynereis dumerilli*	–	–	0.10 ± 0.32	–	–	–
*Spirobranchus* sp.	–	–	0.10 ± 0.32	–	–	–
Syllidae	–	–	–	–	–	0.20 ± 0.63
Arthropoda—8 taxa						
*Balanus* spp	–	–	–	++	++	++
*Cancer pagurus*	0.10 ± 0.32	–	–	–	0.10 ± 0.32	–
*Carcinus maenas*	0.30 ± 0.48 **[5]**	–	0.20 ± 0.42	–	–	0.40 ± 0.52
*Cyathura carinata*	–	0.10 ± 0.32	0.10 ± 0.32	–	–	0.10 ± 0.32
*Leucothoe incisa*	–	–	0.20 ± 0.63	–	–	–
*Melita palmata*	–	–	0.10 ± 0.32	–	–	–
*Porcellana platycheles*	0.10 ± 0.32	0.20 ± 0.42	–	–	–	–
*Sphaeroma* spp	55.20 ± 42.54 **[2]**	–	0.10 ± 0.32	2.75 ± 3.81 **[3]**	0.10 ± 0.32	0.50 ± 1.58
Sipunculidae—1 taxon						
*Golfingia vulgaris*	1.10 ± 1.97 **[3]**	0.10 ± 0.32	0.10 ± 0.32	–	–	–
Cnidaria—1 taxon						
Actiniaria	0.20 ± 0.42	–	–	–	–	–
*Sagartia troglodytes*	–	–	–	0.13 ± 0.35	–	0.10 ± 0.32
Chordata—1 taxon						
Ascididae	–	–	0.20 ± 0.63	–	–	–
Porifera—1 taxon						
Porifera	–	0.20 ± 0.63	–	–	–	–

Bold numbers in brackets are ranks of top five species

*CHAM* Champeaux, *BLSM* Blainville-Sur-Mer, *SGSA* Saint-Germain-Sur-Ay

## Appendix 4

See Table 12.

**Table 12:** List of species and taxa identified during the study

Scientific name	Author	Scientific name	Author
Polychaete		*Nucula hanleyi*	Winckworth, 1931
*Amphitritides gracilis*	(Grube, 1860)	*Ocenebra erinaceus*	(Linnaeus, 1758)
*Aonides oxycephala*	(Sars, 1862)	*Patella intermedia*	Pennant, 1777
*Arabella iricolor*	(Montagu, 1804)	*Patella ulyssiponensis*	Gmelin, 1791
*Boccardia polybranchia*	(Haswell, 1885)	*Patella vulgata*	Linnaeus, 1758
*Branchiomma lucullanum*	Delle Chiaje, 1828	*Ruditapes philippinarum*	(Adams & Reeve, 1850)
*Capitella* sp	Blainville, 1828	*Spisula solida*	(Linnaeus, 1758)
*Cauleriella* sp	(Southern, 1914)	*Trivia monacha*	(da Costa, 1778)
*Cirratulus cirratus*	(O. F. Müller, 1776)	*Venerupis corrugata*	(Gmelin, 1791)
*Eteone longa*	(Fabricius, 1780)	*Venerupis decussata*	(Linnaeus, 1758)
*Eulalia ornata*	Saint-Joseph, 1888		
*Eumida sanguinea*	(Örsted, 1843)	**Arthropoda**	
*Glycera tridactyla*	Schmarda, 1861	*Ampithoe rubricata*	(Montagu, 1818)
*Goniadella bobrezkii*	(Annenkova, 1929)	*Balanus* spp.	Costa, 1778
Hesionidae	**Grube, 1850**	*Cancer pagurus*	Linnaeus, 1758
*Hilbigneris gracilis*	Ehlers, 1868	*Carcinus maenas*	(Linnaeus, 1758)
*Lanice conchilega*	(Pallas, 1766)	*Cheirocratus spp*	Norman, 1867
*Lysidice ninetta*	Audouin & Milne-Edwards, 1833	*Cyathura carinata*	(Krøyer, 1847)
*Lysidice unicornis*	(Audouin & Milne Edwards, 1833)	*Dynamene bidentata*	(Adams, 1800)
*Malacoceros fuliginosus*	(Claparède, 1870)	*Gnathia dentata*	(Sars G.O., 1872)
Maldanidae	Malmgren, 1867	*Idotea granulosa*	Rathke, 1843
*Malmgrenia ljungmani*	(Malmgren, 1867)	*Leucothoe incisa*	(Robertson, 1892)
*Marphysa sanguinea*	(Montagu, 1815)	*Melita palmata*	(Montagu, 1804)
*Nephtys cirrosa*	(Ehlers, 1868)	*Monocorophium acherusicum*	(Costa, 1853)
*Nephtys hombergii*	Savigny in Lamarck, 1818	*Nymphon* sp	Fabricius, 1794
*Nereis caudata*	(Della Chiaje, 1828)	*Orchomene humilis*	(Costa, 1853)
*Notomastus latericeus*	Sars, 1851	*Palaemon elegans*	Rathke, 1837
Paraonidae	**Cerruti, 1909**	*Pilumnus hirtellus*	(Linnaeus, 1761)
*Perinereis cultrifera*	(Grube, 1840)	*Porcellana platycheles*	(Pennant, 1777)
*Pholoe inornata*	Johnston, 1839	Sphaeroma spp	Latreille, 1802
*Phyllodoce laminosa*	Savigny in Lamarck, 1818	*Vaunthompsonia cristata*	Bate, 1858
*Phyllodoce mucosa*	Örsted, 1843		
*Platynereis dumerilii*	(Audouin & Milne Edwards, 1834)	**Chordata**	
Polycirrus medusa	*Grube, 1850*	*Ascidiidae*	***Herdman, 1882***
Sabellaria alveolata	*(Linnaeus, 1767)*	*Blenniidae*	***Rafinesque, 1810***
Schistomeringos neglecta	*(Fauvel, 1923)*		
*Spio* sp	Fabricius, 1785	**Sipuncula**	
*Spirobranchus triqueter*	(Linnaeus, 1758)	*Golfingia (Golfingia) vulgaris*	de Blainville, 1827
*Sthenelais boa*	(Johnston, 1833)		
Syllidae	Grube, 1850	**Nemertea**	
		Nemertea	
**Mollusca**			
*Acanthochitona crinita*	(Pennant, 1777)	Bryozoa	
*Cerastoderma edule*	(Linnaeus, 1758)	*Electra pilosa*	(Linnaeus, 1767)
*Crassostrea gigas*	(Thunberg, 1793)		
*Crepidula fornicata*	(Linnaeus, 1758)	**Cnidaria**	
*Gibbula cineraria*	(Linnaeus, 1758)	Actiniaria	
*Gibbula pennanti*	(Philippi, 1846)	*Sagartia troglodytes*	(Price in Johnston, 1847)
*Gibbula umbilicalis*	(da Costa, 1778)		
*Goodallia triangularis*	(Montagu, 1803)	**Echinodermata**	
Glycymeris glycymeris	(Linnaeus, 1758)	*Amphipholis squamata*	(Delle Chiaje, 1828)
*Lepidochitona cinerea*	(Linnaeus, 1767)		
*Littorina littorea*	(Linnaeus, 1758)	**Insecta**	
*Littorina obtusata*	(Linnaeus, 1758)	*Axelsonia littoralis*	(Moniez, 1890)
*Macoma balthica*	(Linnaeus, 1758)		
*Mytilus edulis*	Linnaeus, 1758	**Porifera**	
*Nassarius reticulatus*	(Linnaeus, 1758)	Porifera	Grant, 1836
*Nucella lapillus*	(Linnaeus, 1758)		
